# Heterogeneities of Site-Specific *N*-Glycosylation in HCC Tumors With Low and High AFP Concentrations

**DOI:** 10.3389/fonc.2020.00496

**Published:** 2020-04-21

**Authors:** Ting Zhao, Li Jia, Jun Li, Chen Ma, Jingyu Wu, Jiechen Shen, Liuyi Dang, Bojing Zhu, Pengfei Li, Yuan Zhi, Rongxia Lan, Yintai Xu, Zhifang Hao, Yichao Chai, Qingshan Li, Liangshuo Hu, Shisheng Sun

**Affiliations:** ^1^College of Life Science, Northwest University, Xi'an, China; ^2^Department of Hepatobiliary Surgery, Institute of Advanced Surgical Technology and Engineering, The First Affiliated Hospital of Xi'an Jiaotong University, Xi'an, China

**Keywords:** hepatocellular carcinoma, alpha-fetoprotein, glycoproteome, intact glycopeptide, site-specific glycosylation, mass spectrometry

## Abstract

Hepatocellular carcinoma (HCC) is still one of the malignant tumors with high morbidity and mortality in China and worldwide. Although alpha-fetoprotein (AFP) as well as core fucosylated AFP-L3 have been widely used as important biomarkers for HCC diagnosis and evaluation, the AFP level shows a huge variation among HCC patient populations. In addition, the AFP level has also been proved to be associated with pathological grade, progression, and survival of HCC patients. Understanding the intrinsic heterogeneities of HCC associated with AFP levels is essential for the molecular mechanism studies of HCC with different AFP levels as well as for the potential early diagnosis and personalized treatment of HCC with AFP negative. In this study, an integrated *N*-glycoproteomic and proteomic analysis of low and high AFP levels of HCC tumors was performed to investigate the intrinsic heterogeneities of site-specific glycosylation associated with different AFP levels of HCC. By large-scale profiling and quantifying more than 4,700 intact *N*-glycopeptides from 20 HCC and 20 paired paracancer samples, we identified many commonly altered site-specific *N*-glycans from HCC tumors regardless of AFP levels, including decreased modifications by oligo-mannose and sialylated bi-antennary glycans, and increased modifications by bisecting glycans. By relative quantifying the intact *N*-glycopeptides between low and high AFP tumor groups, the great heterogeneities of site-specific *N*-glycans between two groups of HCC tumors were also uncovered. We found that several sialylated but not core fucosylated tri-antennary glycans were uniquely increased in low AFP level of HCC tumors, while many core fucosylated bi-antennary or hybrid glycans as well as bisecting glycans were uniquely increased in high AFP tumors. The data provide a valuable resource for future HCC studies regarding the mechanism, heterogeneities and new biomarker discovery.

## Introduction

Hepatocellular carcinoma (HCC), accounting for 90% of primary liver cancer, is the fifth malignant cancer in the world (1). Due to its high morbidity and mortality, HCC is the third leading cause of deaths among cancers worldwide, including ~383,000 deaths each year from HCC in China (2). The main risk factors for HCC include alcoholic liver disease, nonalcoholic steatohepatitis (NASH), chronic hepatitis B virus (HBV) and hepatitis C virus (HCV) infection (1). Although liver surgical resection and transplantation can treat HCC validly, the 5-year overall survival rate is still at ~70% (3).

Early diagnosis and accurate prognosis of HCC are crucial for their effective treatment. Although advances in analytical techniques have produced many novel potential biomarkers, alpha-fetoprotein (AFP) is still the most widely used biomarker for HCC diagnosis and evaluation (4). Unfortunately, the concentration of serum AFP varies dramatically with patients. It is known that 30–40% of HCC patients have a AFP level below the detection level of 20 ng/mL in serum, and remain AFP negative until the later-stage (5–7). Meanwhile, many other studies have also revealed that the high concentration of AFP in patients' serum was associated with high risk of tumor recurrence/progression and reduced survival at different stages of the diseases (8–10). For example, Koji Okuda and co-authors revealed that the level of AFP > 200 ng/mL in serum was the most independently significant factor among several conventional prognostic factors for predicting survival after surgery (11). Bai and co-authors revealed that the AFP level at diagnosis was an independent risk factor of tumor differentiation, TNM stage, tumor size, and survival of HCC patients through 78,743 HCC patients. The study also showed that AFP-positive was associated with less differentiated tumors, more advanced TNM stage, larger tumor sizes, and inferior survival compared with AFP-negative (12). These studies remind us that the AFP levels in HCC patients' sera might be associated with the intrinsic heterogeneities of the HCC tumors. Understanding the molecular mechanism of those heterogeneities associated with AFP levels will be beneficial for the clinical treatment of HCC with different levels of AFP as well as for the new biomarker discovery of HCC patients with AFP negative.

Glycosylation has been recognized as one of the most important post-translational modifications (PTMs) of protein (13, 14). The attached oligosaccharides on proteins are involved in diverse biological processes, such as protein-protein recognition, receptor interaction, cell communication, adhesion, fertilization, embryonic development, immune defense, and inflammation. Abnormal glycosylation has been recognized as an essential feature of tumorigenesis and development including HCC (15). AFP-L3 has been proved by the U.S. Food and Drug Administration (FDA) to be used as another important serum biomarker for HCC diagnosis. It has been confirmed that AFP-L3 has the identical protein sequence as normal AFP but *N*-linked glycans attached at the glycosite Asn-251 of AFP-L3 were core fucosylated (16). The elevated AFP-L3 could further exclude the possibility of other liver diseases such as hepatitis and hepatocirrhosis, and therefore could reduce the false positive results of HCC diagnosed by sole AFP (17). The example of AFP-L3 for HCC diagnosis inspires us to explore the important site-specific glycosylation changes in HCC tumors with various characteristics including different levels of serum AFP.

In this study, we performed integrated glycoproteomic and global proteomic analysis of HCC tumors with low (< 20 ng/mL) and high AFP (> 1,000 ng/mL) in patients' sera to investigate the differences in site-specific glycosylation and proteins between HCC tumors with low and high levels of AFP. The paired paracancer tissues were also included as controls to determine the site-specific glycosylation changes in HCC tumors. The alteration extents in global proteome, site-specific glycosylation and glycosylation occupancy in different HCC samples were systematically compared, and altered site-specific *N*-glycans that commonly or uniquely identified from HCC tumors with low and high AFP were analyzed based on their glycan features and involved Kyoto Encyclopedia of Genes and Genomes (KEGG) pathways. The data will enhance our understanding of HCC heterogeneities that might be associated with AFP concentrations in patients' sera.

## Experimental Section

### Tissue Samples From HCC Patients

Tissue samples were prospectively collected from patient resection with pathological diagnosis of HBV-related hepatocellular carcinoma (HCC) at the First Affiliated Hospital of Xi'an Jiaotong University, China, from 2011 to 2013. The study was approved by Human Ethics Committee at the First Affiliated Hospital of Xi'an Jiaotong University, and written informed consents were obtained from all participants. Based on the AFP concentration, ten tissue samples each with low (<20 ng/mL) and high (>1,000 ng/mL) levels of AFP were selected for this study. The selection of 1,000 ng/mL of AFP as a cutoff was determined based on the Milan criteria of AFP level > 1,000 ng/mL as an exclusion criterion for liver transplantation in patients with HCC (18). The serum AFP concentrations of all patients were measured before surgery, and these patients did not receive any neoadjuvant chemotherapy treatments. The age range of the participants was between 31 and 76 years old, with the average age of 54 ± 11.3. The corresponding paracancer tissues (20 samples) were also collected as control samples. The detailed information of 40 samples (20 pairs) was summarized in (Table S1). Tissue samples were stored at−80 °C until use.

### Protein Digestion

Liver tissues (~ 50mg) were washed twice with PBS (precooled at 4°C) to remove serum. Then tissues were denatured in 8 M urea/1 M NH_4_HCO_3_ buffer, homogenized with a tissue homogenizer (60Hz, Shanghai Jing Xin, China) and sonicated by Ultrasonic Cell Distribution System until the upper solution was clear. The proteins were then reduced by 5mM dithiothreitol (DTT) at 37°C for 1 h and alkylated by 15 mM iodoacetamide at room temperature (RT) in the dark for 30 min. Another 2.5 mM DTT was added and incubated for 10 min at RT. Protein pellets solution was digested by sequencing grade trypsin (protein: enzyme, 100:1; Promega, USA) overnight at 37°C in less than 2M urea/0.25M NH_4_HCO_3_ buffer. The samples were acidified to pH < 2 with trifluoroacetic acid (TFA) and centrifuged at 13,000 rpm for 15 min to remove any particulate matter. The digested peptides were desalted with HLB column (Waters, USA) and eluted with 1 mL solution of 60% (v/v) acetonitrile (ACN) and 0.1% (v/v) TFA. The peptide concentrations were measured by BCA reagent (Beyotime, China).

### Tandem Mass Tag (TMT) Labeling

Equal amounts of tryptic peptides from HCC tumors with low and high AFP, as well as their corresponding paracancerous tissues, were pooled into four samples (10 patients per sample). And they were labeled by four channels of 10-plex TMT reagents (an NH_2_-reactive N-terminal labeling reagent for relative quantitation of intact glycopeptides and protein expressions among samples) according to the manufacturer's protocols (Thermo Fisher Scientific, USA) for protein quantitation (TMT channels: HCC with low AFP and related paracancer: TMT 127N and 126, respectively; HCC with high AFP and related paracancer: TMT 129N and 128C, respectively). The TMT labeled samples were pooled and purified by HLB columns.

### Enrichment of N-Linked Glycopeptides Using Mixed Anion-exchange Extraction Cartridges

The intact glycopeptides were enriched from pooled peptide samples with TMT labels using Mixed Anion-Exchange (MAX) columns. The tryptic peptides mixtures eluted from HLB column (in 60% ACN/0.1% TFA) were diluted by 100% ACN/1% TFA to a final solvent composition of 95%ACN/1%TFA. Peptides were loaded onto MAX extraction cartridges twice and the column was washed three times with 95% ACN/1% TFA. Enriched glycopeptides were eluted in 400 μL of 50% ACN/0.1% FA solution. The samples were dried by vacuum and resuspended in 50 μL of 0.1% FA solution for LC-MS/MS analysis.

### LC-MS/MS Analysis

The TMT-labeled peptide and intact glycopeptide samples were separated by an Easy-nLC™ 1200 system (Thermo Fisher Scientific, Germany) with the use of Acclaim PepMap100 pre-column (2 cm, 75 μm i.d., 3 μm) and Acclaim PepMap100 separating column (50 cm, 75 μm i.d., 3 μm). The mobile phase flow rate was 300 nL/min and consisted of 0.1% FA in water (A) and 0.1% FA in 80% ACN (B). A complete run of 120 min was set as follows: 3–7% B for 1 min, 7-35% B for 90 min, 35–68% B for 19 min, 68–100% B for 1 min and equilibrated in 100% B for 9 min. MS analysis was performed using an Orbitrap Fusion Lumos mass spectrometer (Thermo Fisher Scientific, Germany). The spray voltage was set at 2.4 kV. Orbitrap MS1 spectra (AGC 4 x 10^5^) were collected from 350 to 2000 m/z at a resolution of 60 K followed by data-dependent HCD MS/MS (resolution 50 K, collision energy 37%, activation time 0.1 ms) of the 20 most abundant ions using an isolation width of 1.6 Da. Peptides with charge states from 2 to 8 were selected for MS/MS acquisition. A dynamic exclusion time of 25 s was used to discriminate against previously selected ions.

### Intact Glycopeptide and Protein Identifications

The LC-MS/MS data of global protein from tissue were searched against UniProt human protein databases (downloaded from http://www.uniprot.org on June, 2019) by Sequest in Proteome Discoverer software (Thermo Fisher Scientific, version 2.3). The search parameters for global proteins were set as follows: up to two missed cleavage were allowed for trypsin digestion, 10 ppm and 0.02 Da mass tolerance were set for precursor and MS/MS ions, respectively; Carbamidomethylation (C, +57.021464Da) and 10-plex TMT (N-termini of peptides, +229.162932Da) were set as static modifications; oxidation (M, +15.9949 Da), N-termini acetylation (+42.010565Da), and 10-plex TMT (K at the C-termini of peptides, +229.162932Da) were set as dynamic modifications. All results were filtered with 1% false-discovery rate (FDR). The quantification results from TMT tag were normalized using the normalization factor obtained in proteome data.

The LC-MS/MS data of intact glycopeptides were searched using GPQuest 2.0 (19, 20), with the same parameters as mentioned above for proteomic data using the human UniProt database. For intact glycopeptide identification, the intact *N*-glycopeptide MS data were first converted to “mzML” format using Trans-Proteome Pipline (TPP) and searched against by GPQuest 2.0. The search parameters were set as follows: at least two oxonium ions out of the top 10 fragment ions in the MS/MS spectra were used for extraction of intact glycopeptide MS/MS spectra. The mass tolerances of 10 ppm and 20 ppm were allowed for precursors and fragmentation ions. The FDR of identified intact glycopeptides was estimated by the decoy peptide method and 1% FDR was allowed for intact glycopeptide identification.

### Intact Glycopeptides Quantitation

The quantification information of intact glycopeptides were extracted from their identified MS/MS spectra based on intensities of their TMT reporter ions. The glycopeptide ratios among samples were normalized using the normalization factors obtained from the global proteomic results. After filtering the results by ≥ 5 peptide-spectrum matches (PSMs) per glycopeptide, the medium ratio of each glycopeptide was used for its quantitation.

### Gene Ontology and KEGG Pathway Analyses

Gene ontology (GO) terms enrichment and KEGG pathway analysis were performed using differentially expressed glycoproteins in HCC tumor samples with low and high AFP using STRING: functional protein association networks (https://string-db.org) (21). The thresholds of count > 2 and *p*-value < 0.05 were applied as filters on GO and KEGG pathway analyses of altered proteins.

### Statistical Analysis

Statistical analyses of intact glycopeptide and proteins intensities were performed using SPSS version 22.0. Mean, median, standard deviation and range were calculated for each group of patients. The Mann-Whitney *U*-test was used to calculate *p*-values between AFP-Low and AFP-High tumor diameters. *P*-values less than 0.05 were considered as statistically significant differences and *p*-values less than 0.01 as highly significant difference. Multivariate statistic Principal Component Analysis (PCA) was performed on four pooled samples (low and high AFP tumors, as well as their paracancer tissues, 10 samples per pool) based on their intact glycopeptide expression profiles. PCA of the four pooled samples was created in R 3.2.2 using the FactoMineR (22) (http://CRAN.R-project.org/), which relied on singular value decomposition, and the original feature (intact glycopeptide) space was orthogonally transformed into a set of linearly uncorrelated variables (principal components) (23). Hierarchical clustering analysis of intact glycopeptide, protein and glycosylation occupancy among four pooled samples were performed using QCanvas (http://compbio.sookmyung.ac.kr/~qcanvas) (24).

## Results

### Study Design and the Workflow

**Figure 1:**
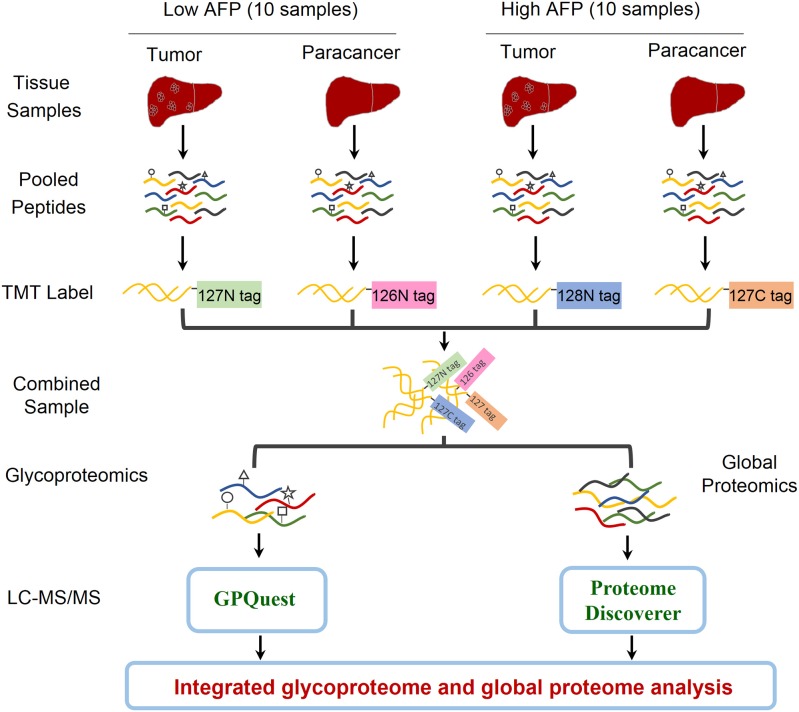
Workflow of integrated glycoproteome and quantitative proteome analysis of hepatocellular carcinoma (HCC) tissues with low and high AFP levels. Proteins were extracted from 20 HCC tumors and 20 paired paracancer tissue samples followed by tryptic digestion. The peptide samples were then pooled into four samples including HCC tumors and paracancer tissues with low and high AFP levels (2 × 2 = 4). After labeling the peptides using the NH2-reactive N-terminal label reagents (10-plex TMT reagents), the peptides were combined and separated into two aliquots. One aliquot was used for intact glycopeptide enrichment and the other aliquot was used for direct proteomic analysis. Both intact glycopeptides and peptides were analyzed by LC-MS/MS and quantitatively identified using GPQuest and Proteome Discoverer for site-specific glycosylation and proteomic analysis, respectively.

In order to explore the heterogeneity of glycosylation in hepatocellular carcinoma (HCC) tissues with different levels of alpha-fetoprotein (AFP) in patient serum, an integrated glycoproteomics and proteomics analysis was performed on two paired groups of HCC-related tissues: ten paired tumor and paracancer tissues with low AFP concentration (< 20 ng/mL) in patients' sera, and ten paired tumor and paracancer tissues with high AFP concentration [> 1,000 ng/mL, an exclusion criterion in Milan Criteria for liver transplantation in patients with HCC (18)] (Figure 1 and Table S1). The whole proteins were extracted from each of 40 tumors/paracancer tissues. The proteins were then digested and pooled into four samples using equal amounts of peptides per sample within each group. Each pooled peptide sample was labeled with one channel of 10-plex TMT reagents. Four TMT-labeled peptide samples were mixed and then separated into two aliquots: one aliquot was used for intact glycopeptides enrichment using mixed anion-exchange (MAX) cartridges for site-specific glycosylation analysis, and the other aliquot was directly analyzed by mass spectrometry for global proteomic analysis. After triplicate LC-MS/MS analyses per sample, intact glycopeptides and proteins were identified and quantified using GPQuest 2.0 and Proteome Discoverer v2.3, respectively. The integrated analyses of glycoproteome and proteome data were then performed to investigate the alterations of site-specific glycans as well as their occupancies. (Figure 1).

### Site-Specific Glycosylation Profiling of HCC Tumors

**Figure 2:**
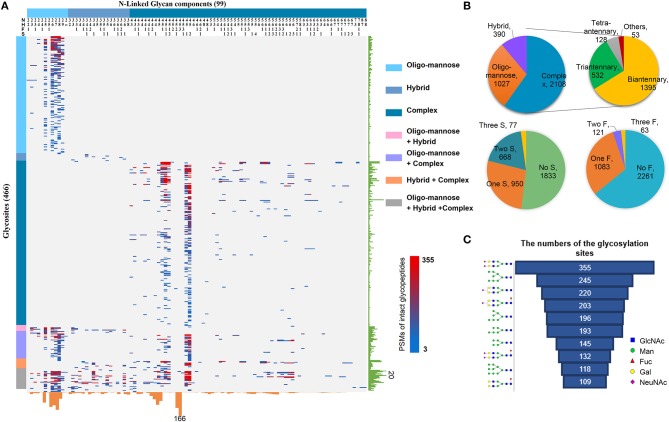
Large-scale profiling of intact glycopeptides in HCC tumors and paired paracancer tissues. **(A)** Profiling of N-linked glycans at individual glycosylation sites identified from all HCC tumor and paracancer samples. The peptide-spectrum-matches (PSMs) of each out of 4,741 intact glycopeptides, comprising of 221*N*-glycans (upper) and 894 glycosites (left), were exhibited in the heat map with different colors. The numbers of glycosites modified by each glycan and of glycans at each glycosite were summarized at the bottom and right parts of the figure, respectively. N: HexNAc; H: Hex; F: Fucose; S: Sialic acid. **(B)** The PSM percentages of different glycans based on glycan subtypes, antennary numbers, as well as the numbers of sialic acid and fucose modifications. **(C)** Top ten glycans detected from all HCC tumor and paracancer tissue samples based on the numbers of their modified glycosylation sites.

By using the above workflow, 4,741 unique N-linked intact glycopeptides were identified from TMT-labeled HCC samples as well as their paracancer tissues within 1% false discovery rate (FDR) (Table S2), which were comprised of 221 *N*-glycan compositions and 1,184 glycosylation sites from 894 glycoproteins (Figure 2A). A representative MS/MS spectrum for intact glycopeptide identification with a peptide YKN#NSDISSTR modified by a core fucosylated glycan HexNAc4Hex5Fuc1Sia1 (N4H5F1S1) is shown in (Figure S1). Among 221 *N*-glycans, 17 were oligo-mannose glycans with seven containing core fucose, 26 were hybrid glycans, and 178 were complex glycans (Figure 2A). As for peptide backbone of the intact glycopeptides, the majority of the glycosites were solely occupied by one kind of glycan subtypes, with 63.5% of which were solely occupied by complex glycans. By calculating the peptide-spectrum matches (PSMs) of intact glycopeptides, we found that the majority of glycosites were occupied by complex glycans (2,108 PSMs, 59.8%), followed by oligo-mannose (1,027 PSMs, 29.1%) and hybrid (390 PSMs, 11.1%) glycans (Figure 2B). Further analysis of complex glycans showed that about 2/3 of complex glycans were biantennary glycans and the other 1/3 were tri/tetra-antennary glycans. In addition, nearly half of the glycosylation sites were modified by glycans containing one to three sialic acids. One third of all glycosites were fucosylated, with 85% of which containing one fucose and the other 15% containing two or more fucoses (Figure 2B).

Based on the numbers of modified glycosylation sites, the top ten glycans from all tumor and paracancer tissue samples were mainly bi-antennary glycans (N4H5S2, N4H5S1, N4H5F1S1, N4H5F1S2, N4H5F1) and oligo-mannose glycans (N2H8, N2H7, N2H6, N2H5, N2H9) (Figure 2C). Except the oligo-mannose glycans that are usually located in the endomembrane system of the cells, the five complex glycans among these top ten glycans are quite similar with the top glycans that were identified from human serum (25), in which biantennary glycans (with 0-1 core fucose and 0-2 sialic acids) were modified on 3/4 of identified glycosites. This is quite reasonable as the serum proteins are mainly synthesized by liver and these complex glycans are normally located on the cell surface and secret glycoproteins.

### Quantitation of Site-Specific Glycosylation in Low and High AFP Level of HCC Tumors

**Figure 3:**
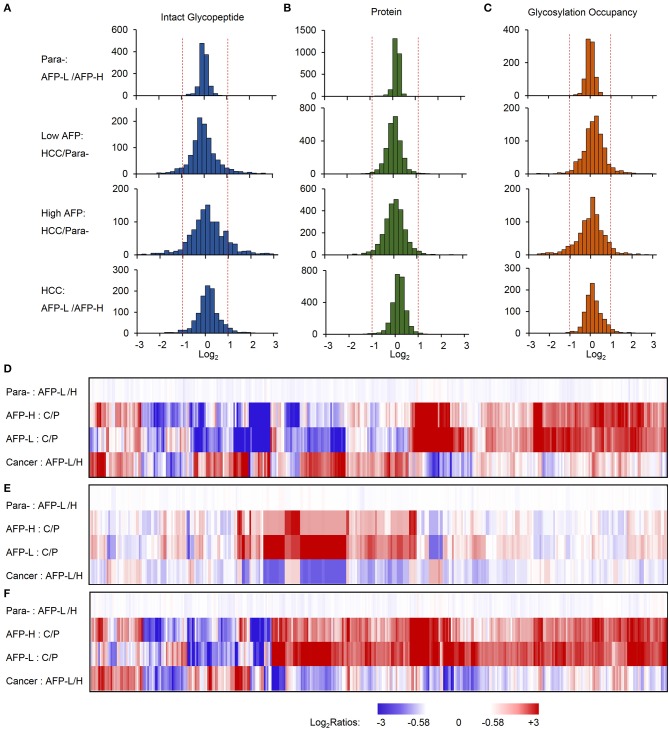
Relative quantification of intact glycopeptides, proteins, and glycosylation occupancies among different sample groups. **(A–C)** The frequencies of intact glycopeptides **(A)**, proteins **(B)**, and glycosylation occupancies **(C)** were compared between paracancer tissues with low and high AFP (upper), low AFP HCC tumors and their paired paracancer tissues, high AFP HCC tumors and their paired paracancer tissues, as well as HCC tumors with low and high AFP (lower). **(D–F)** Heat map shows the fold changes of altered intact glycopeptides **(D)**, corresponding proteins **(E)** and glycosylation occupancies **(F)** among four sample groups. AFP-L: low AFP; AFP-H: high AFP; Para-: paracancer tissue; C/P: cancer/paired paracancer.

The identified intact glycopeptides were then quantified among four groups of samples, including liver tumors and paracancer tissues with low or high level of AFP. To ensure the quantitation accuracy by using the TMT-labeling approach, only intact glycopeptides with at least five MS/MS spectral assignments (1110 glycopeptides) were considered for the quantitation analysis (Table S3). In addition, as almost all glycopeptides (99.9%) were varied within two-fold between two groups of paracancer tissues, we used two-fold change as the cutoff to determine the differentially expressed glycopeptides among samples. Compared with paired paracancer samples, 11.4 and 17.1% of glycopeptides changed in tumors with low and high AFP, respectively (Figure 3A). By further compared the glycopeptide ratios of tumor/paracancer between low and high AFP groups, we identified 82 differently expressed glycopeptides (7.4%) between low and high AFP of HCC tumors.

To further investigate whether these changes occurred at the protein expression level or at the glycosylation occupancies level, we performed integrated analyses of glycoproteome with their corresponding proteome data. At the global proteome level, among 3063 proteins identified from TMT-labeled samples, 99.8% of proteins were varied within two-fold between low and high AFP groups of paracancerous samples, and 98.2 and 95.5% of proteins were changed within 2-fold in low and high AFP tumor samples when compared with their corresponding paracancerous samples, respectively (Figure 3B). The comparison between low and high AFP of tumor samples also showed that 98.7% of proteins varied within 2-fold. These results indicated that the protein expression differences were not as high as the differences of site-specific glycosylation between tumors and paracancerous tissues, as well as between low and high AFP tumors. Next, we calculated the glycosylation occupancy changes in addition to the protein changes by comparing intact glycopeptide ratios with their protein ratios. Using the approach, we found that the glycosylation occupancies on 99.3% of glycopeptides varied within 2-fold between low and high AFP groups of paracancerous samples, but only 90.1 and 82.4% of glycopeptides were stabilized within 2-fold in low and high AFP tumor samples when compared with their corresponding paracancerous samples, respectively (Figure 3C). The comparison between low and high AFP of tumor samples showed that 3.5% of intact glycopeptides changed at glycosylation occupancies. This result was consistent with the difference in glycopeptide levels, clearly indicating that the site-specific glycosylation changes existed not only between HCC tumors and their paired paracancer tissues, but also between low and high AFP tumors, and these differences were much larger at the glycosylation level than that at the protein expression level. This observation was further confirmed by the Principal Component Analysis (PCA) of intact glycopeptides among four groups, which indicated that paracancer tissues with both low and high AFP, low AFP tumors, and high AFP tumors are located in three distinct regions (Figure S2). In addition, a relatively larger difference was observed between high AFP tumors and their paracancer tissues tumors than that in low AFP tumors.

By clustering the intact glycopeptides ratios among four groups of samples, a clear distinction was observed between low and high AFP tumors but no significant difference was observed between two groups of corresponding paracancer samples (Figure 3D and Figure S3), which demonstrated the high heterogeneities of HCC tumors at the site-specific glycosylation level. Comparably, a smaller difference was observed at the overall protein level not only between tumors and paracancer tissues but also between low and high AFP groups of HCC tumors (Figure 3E). Furthermore, the differences among low and high AFP of HCC tumors as well as paracancer tissues were also shown in (Figure 3F), which revealed that many site-specific glycosylation differences among different sample groups occurred at the glycosylation occupancy level instead of the protein expression level.

### Commonly Altered Site-Specific Glycosylation in HCC Tumors

**Figure 4:**
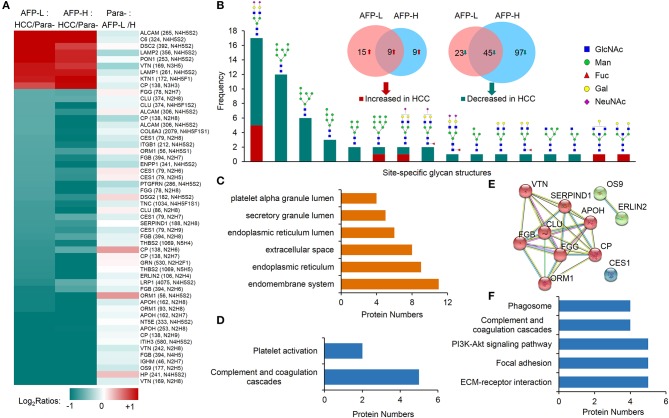
Commonly altered glycopeptides in both low and high AFP of HCC tumors compared with paracancer tissues. **(A)** Profiling of nine increased glycopeptides and 45 decreased glycopeptides in HCC tumors (both low and high AFP). AFP-L: low AFP; AFP-H: high AFP; Para-: paracancer tissues. **(B)** Frequencies of commonly changed site-specific glycans in HCC tumors. **(C)** The cellular components, **(D)** involved pathways, and **(E)** interactions of glycoproteins with decreased oligo-mannose glycan modification in HCC tumors. **(F)** KEGG pathways that involved by glycoproteins with decreased glycosylation in HCC tumors.

We first focused on the investigation of intact glycopeptides that were commonly changed in HCC tumors with both low and high AFP levels. These changes largely represented the site-specific glycosylation alterations in HCC tumors regardless of the AFP concentration in patients' sera. The quantification information of intact glycopeptides were extracted from their identified MS/MS spectra based on intensities of their TMT reporter ions. The glycopeptide ratios among samples were normalized using the normalization factors obtained from the global proteomic results. After filtering the results by ≥5 PSMs per glycopeptide, the medium ratio of each glycopeptide was used for its quantitation. From both low and high AFP-related tumors, we totally identified 54 glycopeptides that had at least 2-fold changes compared with their paracancers, including nine increased and 45 decreased glycopeptides (Figure 4A and Table S4). These glycopeptides were comprised of 17 *N*-glycans and 36 glycosites from 17 glycoproteins. It should be noted that since each glycosite can be modified by multiple glycans, the intact glycopeptide changes only represented the site-specific glycan alterations instead of glycoprotein expression changes. By classifying these changed site-specific glycans based on their attached glycan structures, we found that 25 intact glycopeptides modified by oligo-mannose glycans (Man4–Man9) were decreased in HCC tumors (Figure 4B). These glycopeptides were modified by six oligo-mannose glycans on 15 glycosites from 12 glycoproteins. Eleven of these glycoproteins were located in endomembrane system including nine located in ER (Figure 4C), and five of which were involved in the complement and coagulation cascades pathway (Figure 4D and Table S5A). In addition, a close interaction was observed among eight out of these glycoproteins (Figure 4E). The decrease of oligo-mannose glycan modifications on given glycosites of these glycoproteins might have been involved in tumorigenesis and/or progressions of HCC. Other decreased glycopeptides in HCC tumors included 20 glycopeptides modified by bi-antennary glycans with 0-2 sialic acids, with four of which also containing core fucose (Figure 4B), and two modified by two hybrid glycans. These glycopeptides were identified from 18 glycoproteins, which were mainly involved in complement and coagulation cascades, ECM-receptor interaction, focal adhesion, PI3K-Akt signaling pathway, and phagosome pathways (Figure 4F and Table S5B).

Among nine commonly increased glycopeptides in HCC tumors, six of them were also modified by bi-antennary glycans with 1-2 sialic acids, other three were modified by two bisecting glycans and one Man-9 glycan. These increased glycopeptides were identified from nine glycoproteins, and they were involved in the tuberculosis, phagosome, lysosome, autophagy, and complement and coagulation cascades pathways (Figure S4).

### Heterogeneities of Site-Specific Glycosylation in HCC Tumors With Low and High AFP Concentrations

**Figure 5:**
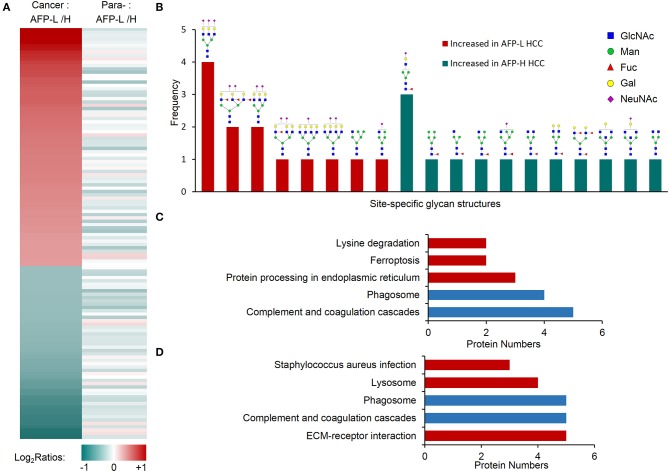
Glycopeptides with increased glycosylation occupancy in either low or high AFP level of HCC tumors. **(A)** Profiling of glycopeptides uniquely increased in low or high AFP of HCC tumors. There were 73 and 53 increased glycopeptides in low and high AFP of HCC tumors, respectively. AFP-L: low AFP; AFP-H: high AFP; Para-: paracancer tissues. **(B)** Frequencies of site-specific glycans that uniquely increased in low or high AFP of HCC tumors. **(C)** KEGG pathways involved by glycoproteins with increased glycosylation in low AFP of HCC tumors. **(D)** KEGG pathways involved by glycoproteins with increased glycosylation in high AFP of HCC tumors. Blue bars represent the common pathways, while red bars represent unique pathways involved by increased glycopeptides in low or high AFP tumors.

We next studied on the intact glycopeptides that show differential expressions in low and high AFP related HCC tumors but didn't show significant differences in their paired paracancer tissues. During the step, the glycosylation occupancy data were used to exclude the effect of possible changes occurred at the protein expression level (related to Figures 3C,F). To get better statistic results, we also reduced our quantification cutoff to a 1.5-fold difference. Using this strategy, we found that 96.9% of intact glycopeptides were varied within the cutoff range between low and high AFP of paracancer samples (Figure 3C). By comparing low and high AFP HCC tumors with each other, we finally identified 67 and 52 increased glycopeptides that belonged to 24 and 39 glycoproteins in low and high AFP of HCC tumors, respectively (Figure 5A and Table S6). These intact glycopeptides didn't show any significant changes in low and high AFP paracancer tissues at either intact glycopeptides, proteins or glycosylation occupancy levels, and therefore should be specific in HCC tumors and associated with AFP concentration in patients' serum.

Some tumor specific glycan subtypes were commonly increased in both low and high AFP tumors compared with their paracancers (Figure S5), which were mainly high-mannose (54/126, 42.8%) and bi-antennary glycans with 1-2 sialic acids (35/126, 27.8%). More interestingly, we found that the glycans uniquely over-expressed in low AFP tumors were mainly tri-antennary with 1-3 sialic acids, two of which also contained one or three antennary fucosylation but no core fucosylation (Figure 5B). The glycans that were uniquely over-expressed in high AFP tumors were mainly core fucosylated glycans (7/10) or bisecting glycans (2/10), most of which were hybrid or bi-antennary complex glycans. These results indicated that the sialylated tri-antennary glycans were uniquely increased in low AFP HCC tumors, while the relatively simple hybrid or bi-antennary glycans with core fucose or bisected HexNAc are uniquely increased in high AFP HCC tumors.

The KEGG pathway enrichment analysis showed that the increased glycoproteins in both low and high AFP tumors were enriched in complement and coagulation cascades, and phagosome pathways (Figures 5C,D, and Table S7). Although these two pathways were also enriched by commonly changed glycoproteins in HCC tumors (related to Figure 4F and Figure S4), these involved glycoproteins were different in different tumor groups (Figure S6), implying that the same pathway could be regulated by different site-specific glycans in different HCC tumor groups. The other pathways that the glycoproteins uniquely increased in low AFP tumors included the “protein processing in endoplasmic reticulum,” “ferroptosis,” and “lysine degradation,” the glycoproteins uniquely increased in high AFP tumors involved included “ECM-receptor interaction,” “lysosome,” and “staphylococcus aureus infection” (Figures 5C,D).

## Discussions

HCC is one of the malignant tumors with high morbidity and mortality, which places a heavy burden on society. AFP is a widely used serum biomarker for detecting liver cancer worldwide. The *N*-glycosylated isoform of AFP (AFP-L3), which contains core fucosylation on its N-linked glycans, only exists in HCC but is absent in other liver diseases and therefore can further enhance the specificity of HCC diagnosis. Unfortunately, a quite large proportion of HCC patients have an AFP negative until the later-stage (5, 6). In addition, the AFP level has also been associated with pathological grade, progression, and survival HCC patients. Understanding the intrinsic heterogeneities of HCC associated with AFP levels will be beneficial for the diagnosis and clinical treatment of HCC patients with AFP negative.

Due to the important roles of protein glycosylation on the regulation of protein structures and functions, glycoproteomic analysis of HCC has been reported in several previous studies (26–29). In these studies, the commonly used analytical strategy was to separate the glycans from the glycosylated peptides by enzymatic or chemical methods, and then separately performed mass spectrometry analysis on the released glycan or deglycosylated peptide. The overall expression and variation of glycans and/or glycoproteins were analyzed in the samples. This strategy has been well applied in liver cancer related research, but due to the heterogeneity of glycosylation of proteins, complete information about glycosylation sites and their corresponding glycans couldn't be obtained using this strategy (30). With the rapid development of mass spectrometry and the advancement of related analytical methods, directly intact glycopeptide analysis has been achieved in recent years (25, 31, 32). These methods and tools give us additional opportunities for heterogeneity analysis of site-specific glycosylation in HCC.

In this study, we first profiled site-specific glycans from low and high AFP levels of HCC tumors and non-tumor tissues. Overall, 4,741 unique N-linked intact glycopeptides were identified and quantified, containing 221 *N*-glycan compositions modified on 1,184 glycosylation sites from 894 glycoproteins. Nearly half of these glycosylation sites were modified by sialylated glycans, which indicated that sialylation in liver cancer tissues was relatively common. Sialylation is an important modification in cellular glycosylation, as sialylated carbohydrates have an important role in cellular recognition, cell adhesion and cell signaling (33). Especially the increased level of α2,6- and α2,3- linked sialylation is closely related to cancer (34). In addition, 1/3 of the complex glycan modifications are tri/tetra-antennary or more branched glycans. Increased number of branches on N-linked glycan have been shown to be associated with liver cancers (17, 35). Unlike the previously reported studies of serum glycosylation where sialylated biantennary glycans were the main glycans, high mannose glycans also accounted for a large proportion of *N*-glycans in tissues. This is due to the fact that during the synthesis of *N*-glycans, high mannose is mainly in the Golgi and endoplasmic reticulum (intracellular), while complex glycans are mainly extracellular (36).

The investigation of intrinsic heterogeneities of the HCC tumors with different AFP levels is the first step toward the molecular mechanism exploration of AFP level differences in HCC tumors. In previous studies, glycomics and immunohistochemical staining approaches have been applied in the quantitative analysis of released glycans from glycoproteins in HCC serum (37–40). Many altered glycans, including sialylated, bisecting, and core fucosylated glycans, had been identified in HCC serum, but few study focused on the glycan heterogeneity analysis of HCC tumors with different AFP levels. In a recent study, Jiang and co-authors have reported the great differences of proteomes between low and high AFP of HCC tumors (41), which inspired us to explore the site-specific glycosylation differences in HCC tumors with different levels of serum AFP by using our recently developed approach for intact glycopeptide analysis (42). In this study, by integrating quantitative information from the proteome and glycoproteome, large numbers of glycopeptides that specifically elevated in low or high AFP level of HCC tumors were identified. By comparing the quantification data between glycoproteome with global proteome, we found that the differences between low and high AFP of HCC tumors were much greater at the site-specific glycosylation level than at the protein expression level. In addition, by further analyzing the glycan structures on these glycopeptides, we interestingly found for the first time that several sialylated but not core fucosylated tri-antennary glycans were uniquely increased in low AFP tumors, while a large numbers of core fucosylated bi-antennary or hybrid glycans or bisecting glycans were uniquely increased in high AFP tumors. These data revealed the high heterogeneities of site-specific glycans between low and high AFP level of HCC tumors, even though their functional implications still need further investigations.

In summary, an integrated glycoproteomic and proteomic analysis of low and high AFP level of HCC tumors was performed in this study to explore the intrinsic heterogeneities of site-specific glycosylation associated with different AFP levels of HCC. In addition to the profiling the site-specific glycosylation in HCC tumors, we found that the differences between low and high AFP of HCC tumors were much greater at the site-specific glycosylation level than the protein expression level. By relative quantifying the intact glycopeptides among four sample groups, we uncovered the great heterogeneities of site-specific *N*-glycans between HCC tumors and non-tumors as well as between low and high AFP level of HCC tumors. The data provide a valuable resource for various HCC researches including new biomarker discovery of HCC with AFP-negative, the personalized treatment of HCC, as well as the carcinogenesis of HCC.

## Data Availability Statement

The mass spectrometry data have been deposited to the ProteomeXchange Consortium (http://proteomecentral.proteomexchange.org) via the PRIDE partner repository with the dataset identifier PXD016406.

## Ethics Statement

The studies involving human participants were reviewed and approved by Human Ethics Committee at the First Affiliated Hospital of Xi'an Jiaotong University, Xi'an, China. The patients/participants provided their written informed consent to participate in this study.

## Author Contributions

TZ, LJ, and SS designed the experiments. YC, QL and LH collected the tissue samples and clinical information. TZ performed experiments with help from LD, BZ, YZ, RL, and ZH. TZ performed MS analysis. TZ analyzed data with the help from JL, CM, JW, JS, PL, and YX. TZ, JL, and SS wrote and edited the manuscript, all the authors contributed to manuscript review.

## Conflict of Interest

The authors declare that the research was conducted in the absence of any commercial or financial relationships that could be construed as a potential conflict of interest.

## Abbreviations

HCC: Hepatocellular Carcinoma
AFP: Alpha-fetoprotein
NASH: Nonalcoholic Steatohepatitis
HBV: Chronic Hepatitis B Virus
HCV: Chronic Hepatitis C Virus
PSM: Peptide-spectrum Match
GO: Gene Ontology
KEGG: Kyoto Encyclopedia of Genes and Genomes
PCA: Principal Component Analysis
FDR: False Discover Rate.
