# Supplementary material for: Heterogeneities of Site-Specific N-Glycosylation in HCC Tumors With Low and High AFP Concentrations
Source: Front Oncol. 2020 Apr 21;10:496. doi: 10.3389/fonc.2020.00496 (PMC7212448; doi:10.3389/fonc.2020.00496)
Supplement: Supplementary file 1 [file Data_Sheet_1.PDF]

Supplementary Material

1 Supplementary Tables

Table S1 listing the clinical information of HCC and paracancer tissue samples used in this study.

Table S2 listing all identified intact glycopeptides from HCC samples;

Table S3 listing quantitative intact glycopeptides in all HCC tumors and paired paracancer tissues.

Table S4 listing commonly altered intact glycopeptides in both low and high AFP of HCC tumors.

Table S5 listing KEGG pathways that were involved by glycoproteins with commonly decreased glycans in both low and high APF of HCC tumors.

Table S6 listing uniquely increased intact glycopeptides in either low or high AFP of HCC tumors.

Table S7 listing KEGG pathways that were involved by glycoproteins with uniquely increased glycans in low and high APF of HCC tumors.

2 Supplementary Figures

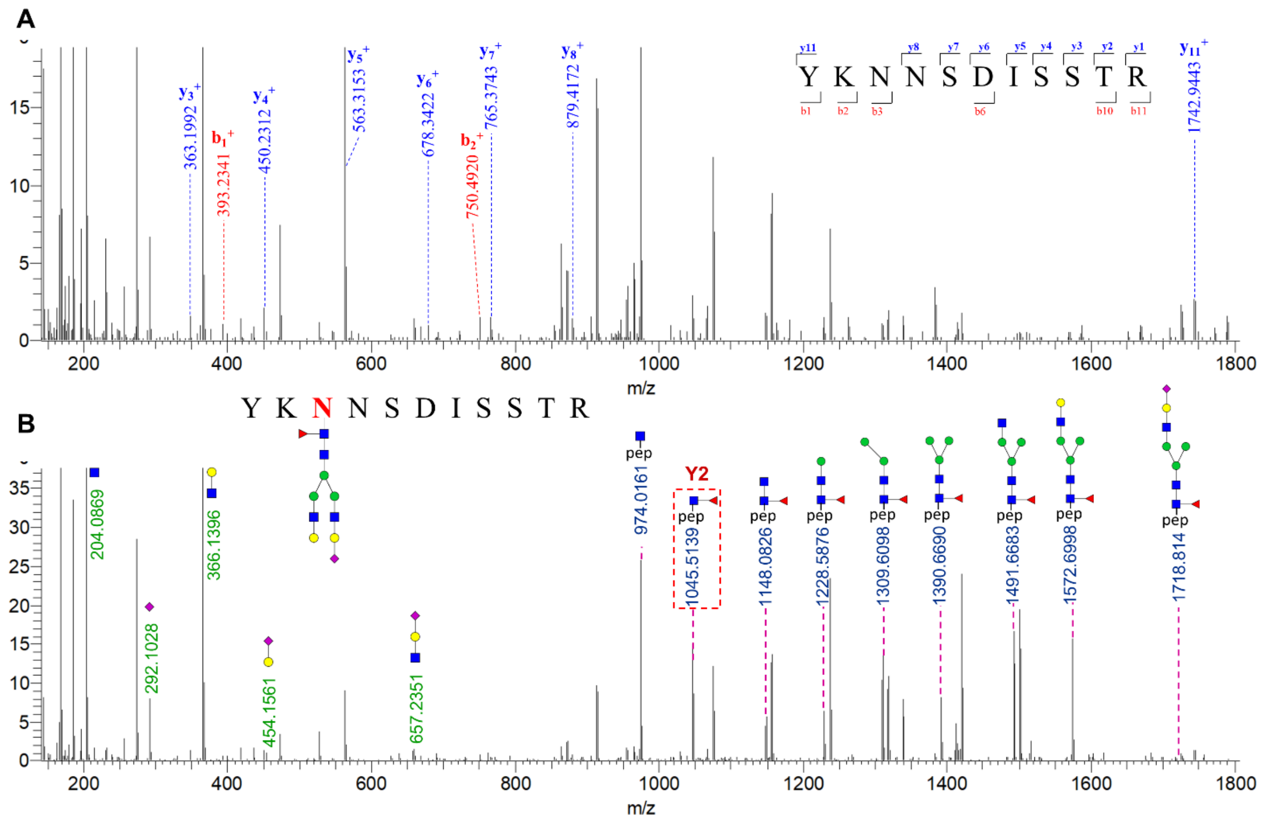

Figure S1. A representative MS/MS spectrum for identification of an intact glycopeptide with peptide YKN#NSDISSTR modified by a core fucosylated glycan HexNAc4Hex5Fuc1Sia1 (N4H5F1S1) from immunoglobulin heavy constant mu (IGHM). The intact glycopeptide was fragmented by two individual HCD energies (HCD=33 and HCD=20) in one LC-MS/MS analysis. (A) The sequence of the peptide was identified by matched b and y ions from high HCD energy of MS/MS spectrum (HCD=33). #indicates the glycosylation site. (B) The glycan was identified from low HCD energy of MS/MS spectra (HCD=20), and core fucosylation was identified by five feature Y ions (from peptide+$HexNAc_1Fuc_1$ ion at m/z=1045.51 to peptide+$HexNAc_2Hex_3Fuc_1$ ion at m/z=1390.67). The main feature Y2 ion is marked in red box. The m/z values with charge states 1+ and 2+ are labeled by green and blue, respectively.

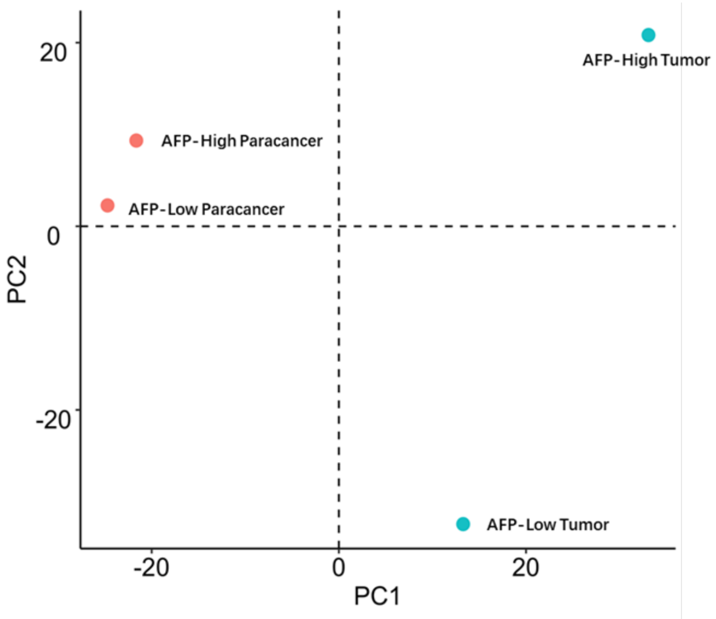

Figure S2. Principal Component Analysis of intact glycopeptides identified from low and high AFP tumors as well as their paired paracancer tissues. The PCA results showed that paracancer tissues (low and high AFP paracancer groups were adjacent), low AFP tumors, and high AFP tumors are located at three distinct regions.

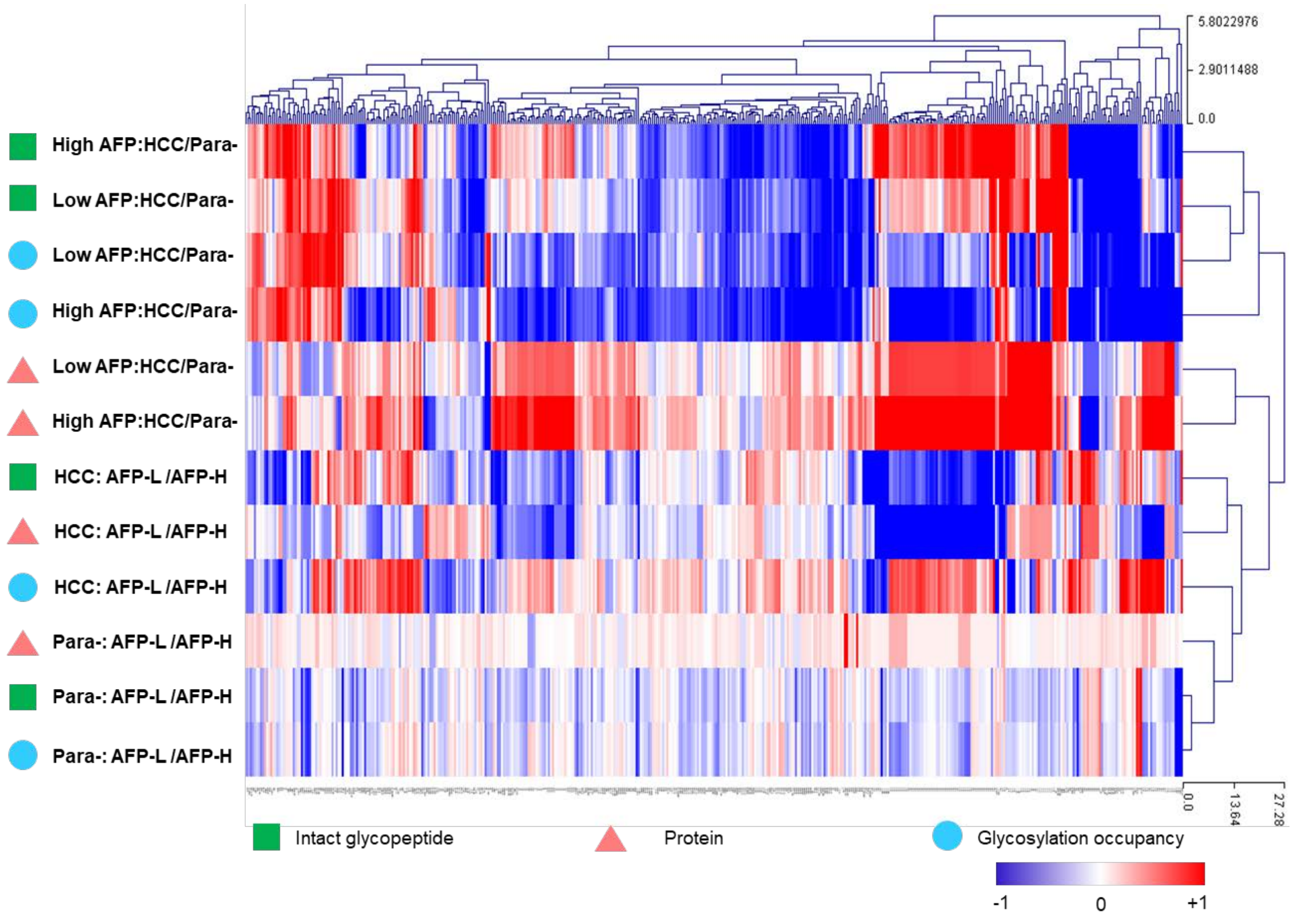

Figure S3. Two-way clustering of low AFP tumors, high AFP tumors, as well as their paired paracancer tissues based on their intact glycopeptide ratios. This Figure is related to Figure 3D-3F. AFP-L: AFP-low; AFP-H: AFP-high; Para-: Paracancer; HCC/Para-: Cancer/Paracancer.

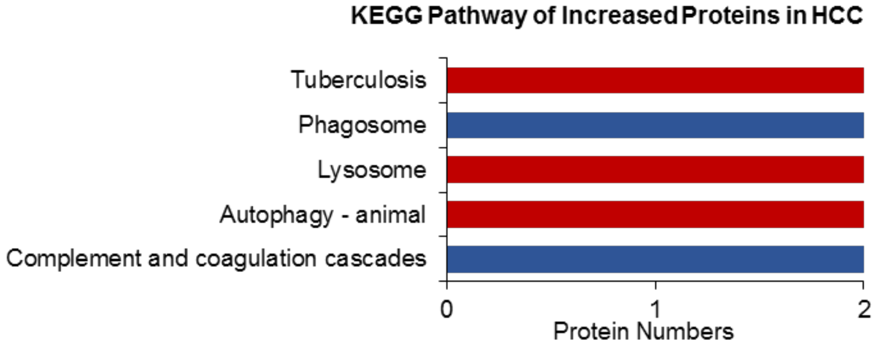

Figure S4. Kyoto Encyclopedia of Genes and Genomes (KEGG) pathway that involved by glycoproteins modified by commonly increased site-specific glycans in both low and high AFP level of HCC tumors. Red represents the unique pathways involved by commonly increased glycopeptides in both low and high AFP level of HCC tumors, while blue represents the pathways that were also involved by the uniquely increased in either low or high AFP level of HCC tumors, which related to the Figure 5C and 5D in the manuscript.

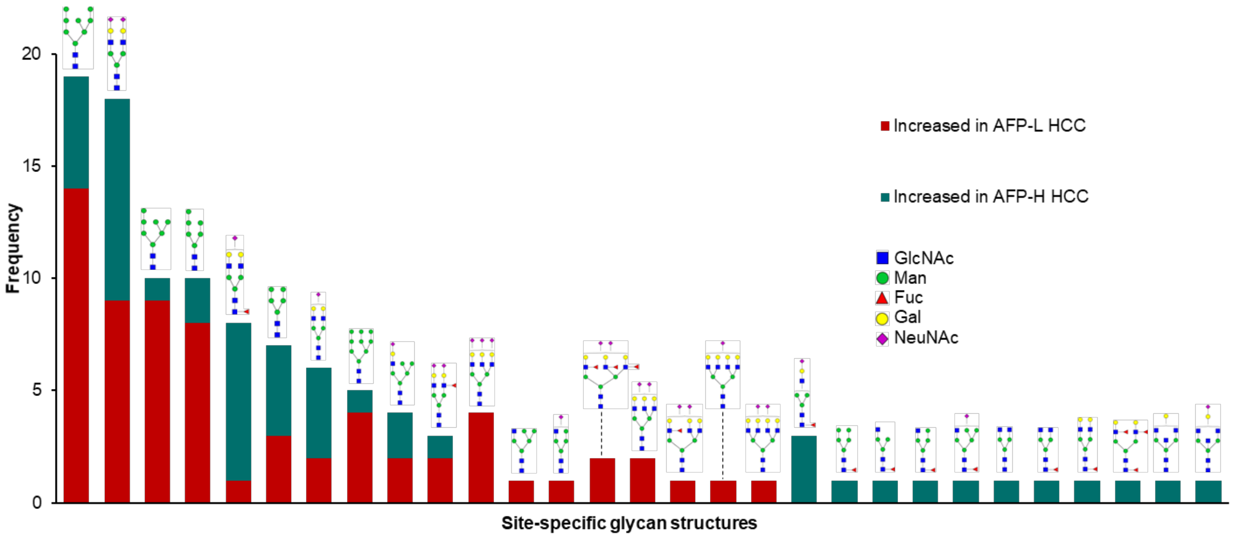

Figure S5. Frequency of site-specific glycans that specificly increased in low or high AFP of HCC tumors.

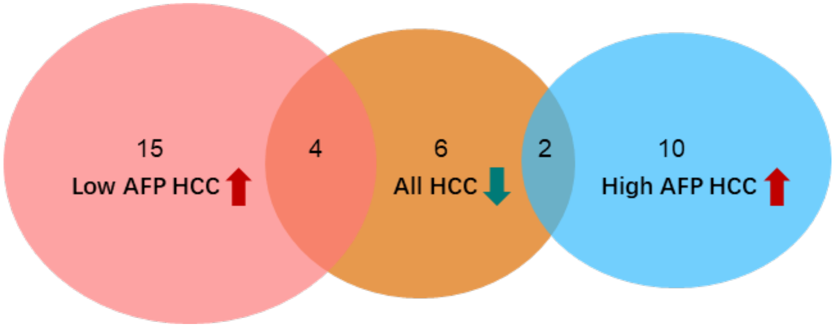

KEGG Pathway	Common proteins	HCC Tumor	Low AFP HCC Tumor	High AFP HCC Tumor
Complement and coagulation cascades	CLU	N2H8, N4H5F1S2	N2H6, N2H8, N2H7, N4H5S2, N5H6S3, N5H6S2, N5H6F3S2, N5H6F1S2, N5H6S2	N4H5F1S1, N4H5F1S2
	FGB	N2H7, N4H5	None	N2H7, N2H8, N4H5F1S1, N4H5F3
	FGG	N2H6, N2H7, N2H8	N4H5S2	N4H5F1S1
	VTN	N2H8, N3H5	N2H5, N2H7, N2H8, N3H4S1, N3H5, N3H6S1, N4H5F1S2, N4H5S1, N4H5S2	None
Phagosome	LAMP1	N4H5S2	N2H5, N2H6, N2H7	N3H4F1S1
	LAMP2	N4H5S2	N6H7S1, N6H7S2, N4H5S2, N5H6S3	N3H4F1S1
	THBS2	N5H4 ,	None	N5H4S1

Figure S6. Comparison of glycoproteins that involved in the same KEGG pathways but their site-specific glycans were commonly or uniquely increased in low and high APF levels of HCC tumors.
